# Deoxynivalenol Impairs Porcine Intestinal Host Defense Peptide Expression in Weaned Piglets and IPEC-J2 Cells

**DOI:** 10.3390/toxins10120541

**Published:** 2018-12-15

**Authors:** Shuai Wang, Jiacheng Yang, Beiyu Zhang, Kuntan Wu, Ao Yang, Chong Li, Jiacai Zhang, Cong Zhang, Shahid Ali Rajput, Niya Zhang, Lvhui Sun, Desheng Qi

**Affiliations:** Department of Animal Nutrition and Feed Science, College of Animal Science and Technology, Huazhong Agricultural University, Wuhan 430070, China; wangshuai@mail.hzau.edu.cn (S.W.); yangjiacheng@webmail.hzau.edu.cn (J.Y.); zhangbeiyu@webmail.hzau.edu.cn (B.Z.); kuntanwu@webmail.hzau.edu.cn (K.W.); yangao@webmail.hzau.edu.cn (A.Y.); chrisli@webmail.hzau.edu.cn (C.L.); zjc404@webmail.hzau.edu.cn (J.Z.); zhangcong@webmail.hzau.edu.cn (C.Z.); dr.shahidali@hotmail.com (S.A.R.); zhangniya@mail.hzau.edu.cn (N.Z.); lvhuisun@mail.hzau.edu.cn (L.S.)

**Keywords:** Deoxynivalenol, Weaned piglets, Host defense peptides, NOD2, Caspase-12

## Abstract

Host defense peptides (HDPs) are efficient defense components of the innate immune system, playing critical roles in intestinal homeostasis and protection against pathogens. This study aims to investigate the interference effects of DON on the intestinal porcine HDPs expression in piglets and intestinal porcine epithelial cell line (IPEC-J2) cells, and elucidate the underlying mechanisms through which it functions. In an animal experiment, intestinal HDPs were determined in weaned piglets fed control and 1.28 mg/kg or 2.89 mg/kg DON-contaminated diets. Dietary exposure to DON significantly decreased piglet average daily gain, increased intestinal permeability and depressed the expression of porcine β-defensin1 (pBD1), pBD2, pBD3, epididymis protein 2 splicing variant C (pEP2C), PMAP23, and proline/arginine-rich peptide of 39 amino acids (PR39) in the intestine (*p* < 0.05). In IPEC-J2 cells, DON decreased cell viability and inhibited the expression of pBD1, pBD3, pEP2C, PG1-5, and PR39 (*p* < 0.05). NOD2, key regulator that is responsible for HDPs production, was markedly downregulated, whereas caspase-12 was activated in the presence of DON. In conclusion, DON induced caspase-12 activation and inhibited the NOD2-mediated HDPs production, which led to an impaired intestinal barrier integrity of weaned piglets. Our study provides a promising target for future therapeutic strategies to prevent the adverse effects of DON.

## 1. Introduction

Deoxynivalenol (DON) is one of the most prevalent trichothecene mycotoxin generated from *Fusarium* species and is commonly detected in cereals, such as wheat, corn, oats, rye and barley, all over the world [1]. Among domestic animals, swine exhibit the highest sensitivity to DON. Consumption of diets contaminated with DON at ≥0.9 mg/kg causes anorexia, nutrient malabsorption, reduced weight gain, and immunologic alterations in pigs, especially for piglets [2]. Following ingestion of contaminated food or feed, DON is mainly absorbed in the intestine. The intestinal tract is the primary target for DON. Recent work has demonstrated that noncytotoxic concentration of DON significantly downregulated the porcine β-defensin 1 (pBD-1) mRNA expression in porcine jejunal epithelial cells [3]. As a key component of the innate immune system, host defense peptides (HDPs) serve a fundamental role in maintaining mucosal homeostasis and fighting against infections [4,5]. Defensins and cathelicidins represent two major families of HDPs in vertebrates, which are produced mainly by mucosal epithelial cells and phagocytes [6,7]. Dysregulation of defensin production has been found in diverse models of intestinal diseases and inflammation [8,9]. 

Pigs are very sensitive to DON, and could be effortlessly exposed to this toxin via their cereal-based diets. However, the mechanisms of DON in disturbing the expression of intestinal HDPs in piglets are currently poorly understood. NOD2 is a member of the nucleotide-binding domain, leucine-rich repeat (NLR) family of cytosolic proteins involved in intracellular recognition of bacteria by sensing muramyl dipeptide (MDP) [10]. Emerging evidence suggests that NOD2 is necessary for the production of essential intestinal host defense peptides, specifically defensins [11,12]. NOD2 mutations and impaired defensin expression have been linked to inflammatory bowel diseases [13].

Caspases are indispensable for both apoptosis and inflammatory cytokines processing [14]. Caspase-12 is predominantly localized in endoplasmic reticulum (ER) and is specifically irritated by ER stress [15]. It has been reported that caspase-12 could inhibit the NOD signaling and dampen the downstream HDPs production [16], whereas caspase-12 deficiency facilitates HDPs production as well as consequently enhancing bacterial clearance and disease resistance in mice [16,17].

Research has found DON exposure can evoke ER stress in murine peritoneal macrophage [18]. Meanwhile, Park et al. [19] revealed that in human enterocytes, NOD2-induced chemokine production was suppressed markedly in response to DON. Therefore, we hypothesize that the NOD2/caspase-12 pathway is critical for DON-induced dysregulation of HDPs. Identifying the mechanism of DON in disturbing the expression of intestinal HDPs can provide a promising target for future therapeutic strategies. The aim of this investigation was to assess the effects of DON on porcine intestinal HDPs expression *in vivo* and *in vitro*, and elucidate the underlying mechanisms through which it functions.

## 2. Results

### 2.1. Piglet Performance

As shown in Table 1, the body weight of pigs in 1.28 mg/kg DON group was lower (*p* < 0.05) than that of the control on d 14 and 28. In addition, pigs fed 2.89 mg/kg DON-contaminated diet also had lower (*p* < 0.01) body weight than those in the control treatment on d 28. The average daily gain (ADG) was significantly decreased (*p* < 0.05) in 2.89 mg/kg DON treated pigs compared to the control piglets during d 14 to 28, and d 1 to 28. Treatment with both two DON levels led to significant decreased (*p* < 0.05) average daily feed intake (ADFI) during 1 to 14, d 15 to 28, and d 1 to 28. No significant difference was observed among control, 1.28 mg/kg DON group, and 2.89 mg/kg DON group with regard to gain: feed (G:F). Moreover, diarrhea incidence and diarrhea scores of piglets in these two levels of DON treatments were higher than those in the control.

### 2.2. Intestinal Morphology

**Figure 1:**
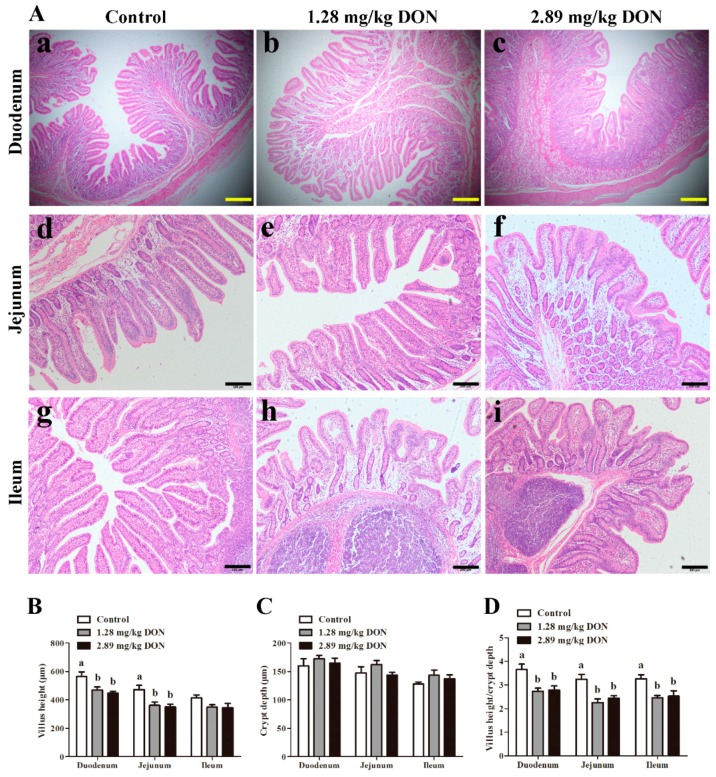
Effect of oral deoxynivalenol (DON) exposure on small intestinal morphology. (**A**) Representative photomicrographs of villus in duodenum, jejunum and ileum from control, piglets fed 1.28 mg/kg or 2.89 mg/kg DON-contaminated diet. (**a**–**c**) villus in duodenum from control, piglets fed 1.28 mg/kg or 2.89 mg/kg DON-contaminated diet, respectively; (**d**–**f**) villus in jejunum from control, piglets fed 1.28 mg/kg or 2.89 mg/kg DON-contaminated diet, respectively; (**g**–**h**) villus in ileum from control, piglets fed 1.28 mg/kg or 2.89 mg/kg DON-contaminated diet, respectively. The yellow bar represents 500 μm, and the black bar represents 200 μm. (**B**) Villi height, (**C**) crypt depth, and (**D**) villi height to crypt depth of duodenum, jejunum, and ileum in response to DON exposure. Pigs were fed a control diet (□) or a diet contaminated with 1.28 mg DON/kg feed (■), and 2.89 mg DON/kg feed (■). Values are means ± SEM, n = 8. ^a,b^ Mean values without a common letter were significantly different (*p* < 0.05).

The intestinal mucosa in the control group showed normal morphology (Figure 1). However, piglets fed with the diets contaminated with DON at 1.28 mg/kg and 2.89 mg/kg levels exhibited slight to modest intestinal lesions. The major histological changes detected were villi fusion and atrophy, diffuse apical necrosis, and flattened enterocytes. 

Alternation of villus height reflects disturbance in the balance between epithelial cell proliferation and apoptosis. As shown in Figure 1B, animals that ingested 1.28 mg/kg or 2.89 mg/kg DON-contaminated diet had lower (*p* < 0.05) villus height in duodenum and jejunum than that of the control pigs. No significant change was observed in crypt depth in any intestinal region (Figure 1C). However, villus height to crypt depth ratio decreased (*p* < 0.05) significantly in all regions of the intestine of piglets that received 1.28 mg/kg or 2.89 mg/kg DON-contaminated diet (Figure 1D).

### 2.3. Intestinal Permeability

**Figure 2:**
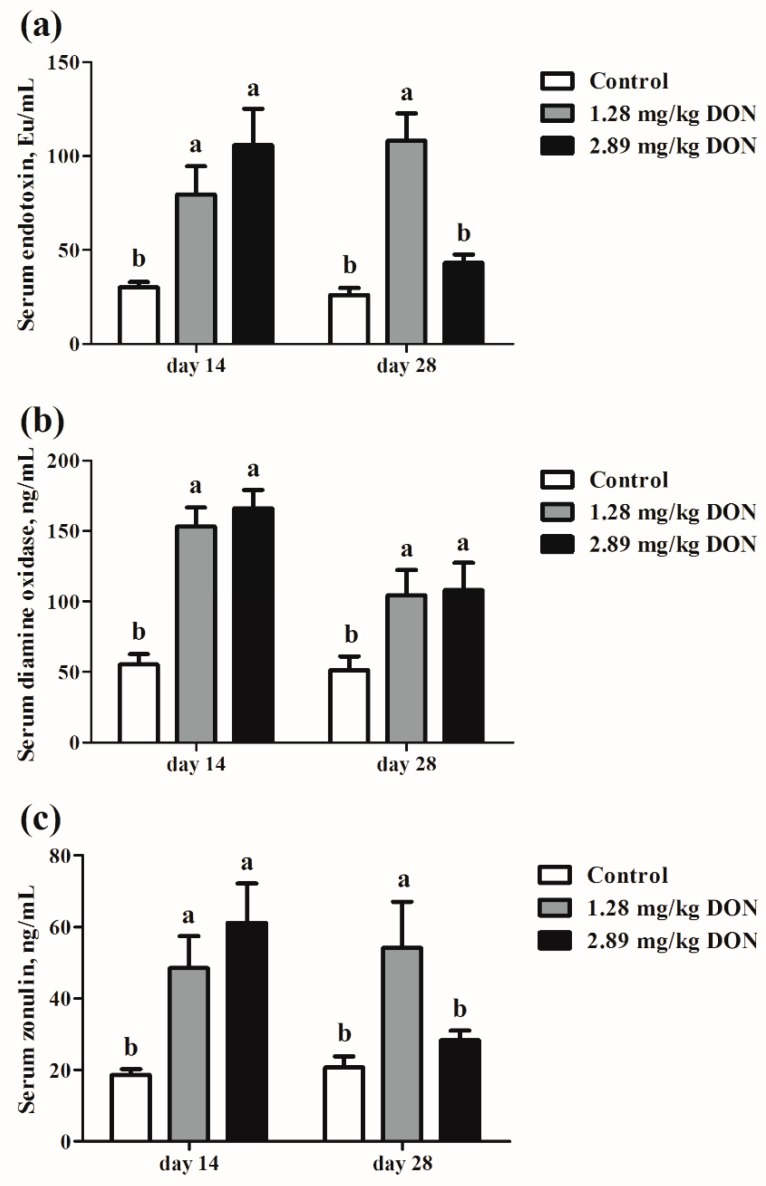
Effect of deoxynivalenol (DON) exposure on serum endotoxin (**A**), diamine oxidase (**B**), and zonulin (**C**) contents of weaned piglets. Pigs were fed a control diet (□) or a diet contaminated with 1.28 mg DON/kg feed (■), and 2.89 mg DON/kg feed (■). Values are means ± SEM, n = 8. Within the same day, ^a,b^ Mean values without a common letter were significantly different (*p* < 0.05).

Based on the aforementioned experiments, three biomarkers associated with intestinal permeability were analyzed. On d 14, the serum levels of endotoxin, diamine oxidase, and zonulin were higher (*p* < 0.05) in piglets ingested 1.28 mg/kg or 2.89 mg/kg DON-contaminated diet than those fed the control diet (Figure 2). On d 28, 1.28 mg/kg DON treatment significantly increased (*p* < 0.05) the serum concentrations of endotoxin, diamine oxidase, as well as zonulin compared with the control group. Additionally, animals that ingested 2.89 mg/kg DON-contaminated had greater (*p* < 0.05) serum diamine oxidase concentration than those that were fed the control diet (Figure 2B). Prior investigations have demonstrated that the impaired intestinal barrier integrity could increase content of endotoxin as well as enhance concentrations of diamine oxidase and zonulin in blood [20,21]. These results indicate that DON exposure increases the intestinal permeability in weaned piglets.

### 2.4. The Effect of DON on Porcine Intestinal HDPs Expression in Weaned Piglets

**Figure 3:**
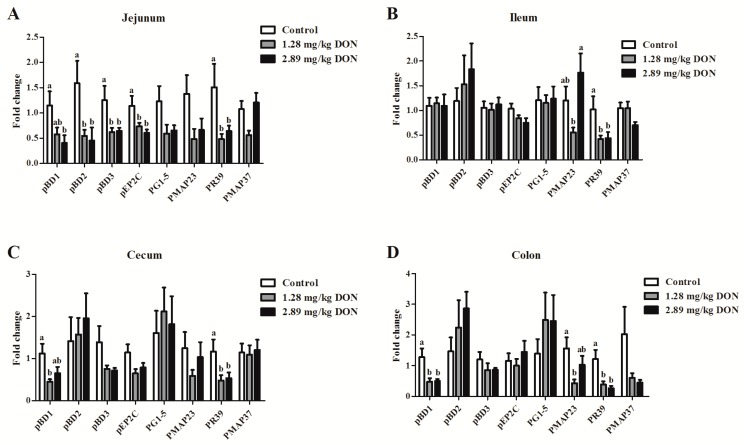
Effect of deoxynivalenol (DON) exposure on the jejunum (**A**), ileum (**B**), cecum (**C**), and colon (**D**) mRNA expression of host defense peptides. Pigs were fed a control diet (□) or a diet contaminated with 1.28 mg DON/kg feed (■), and 2.89 mg DON/kg feed (■). Values are means ± SEM, n = 8. ^a,b^ Mean values without a common letter were significantly different (*p* < 0.05).

**Figure 4:**
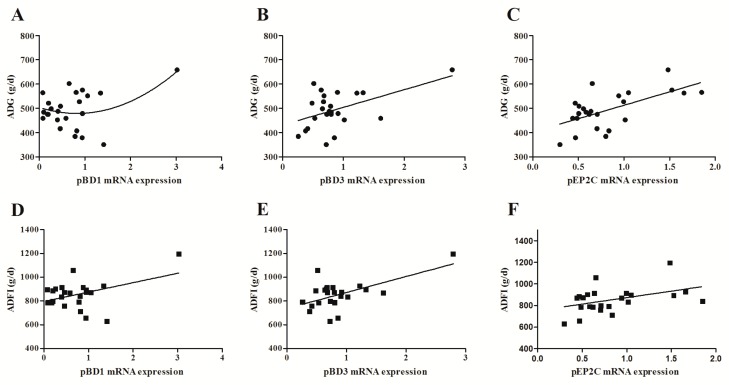
Regression relationships between piglet performance parameters and mRNA expression levels of host defense peptide genes in the jejunum of piglets. Regression relationships between average daily gain (ADG) in week 4 and the mRNA levels of (**A**) pBD1 (y = 36.047x^2^ − 59.92x + 504.12; R^2^ = 0.21; *p* = 0.08), (**B**) pBD3 (y = 72.901x + 431.19; R^2^ = 0.25; *p* = 0.01), and (**C**) pEP2C (y = 110.71x + 402.12; R^2^ = 0.38; *p* < 0.01) genes in the jejunum of piglets. Regression relationships between average daily feed intake (ADFI) in week 4 and the mRNA levels of (**D**) pBD1 (y = 79.084x + 795.93; R^2^ = 0.178; *p* = 0.04), (**E**) pBD3 (y = 134.23x + 737.68; R^2^ = 0.34; *p* < 0.01), and (**F**) pEP2C (y = 119.56x + 753.62; R^2^ = 0.18; *p* = 0.04) genes in the jejunum of piglets.

The results presented in Figure 3 show that the mRNA expression of pBD1, pBD2, pBD3, pEP2C, and PR39 was significantly down-regulated (*p* < 0.05) in jejunum of piglets fed 1.28 mg/kg or 2.89 mg/kg DON-contaminated diet compared to the control pigs. Ileal PMAP23 and PR39 mRNA levels were lower (*p* < 0.05) in the 1.28 mg/kg or 2.89 mg/kg DON-treated group than the control group. Compared with the control group, DON exposure significantly decreased (*p* < 0.05) the relative abundances of mRNA for pBD1 and PR39 in the cecum. Moreover, the colonic pBD1, PMAP23 and PR39 mRNA levels were also down-regulated (*p* < 0.05) in piglets that received the 1.28 mg/kg or 2.89 mg/kg DON-contaminated diet compared to the control piglets. Further regression analysis showed that ADG was tended to be positively (*p* = 0.08) associated with jejunal pBD1 mRNA levels (Figure 4A). ADG linearly decreased (*p* < 0.05) with a decline in jejunal pBD3 and pEP2C mRNA levels (Figure 4B,C). In addition, a significant linear relationship (*p* < 0.05) between ADFI and the mRNA levels of pBD1, pBD3, and pEP2C was also observed. ADFI was decreased (*p* < 0.05) linearly with the decrease in jejunal pBD1, pBD3, and pEP2C mRNA levels (Figure 4D–E). 

### 2.5. Activation of Caspase-12 in Jejunum 

**Figure 5:**
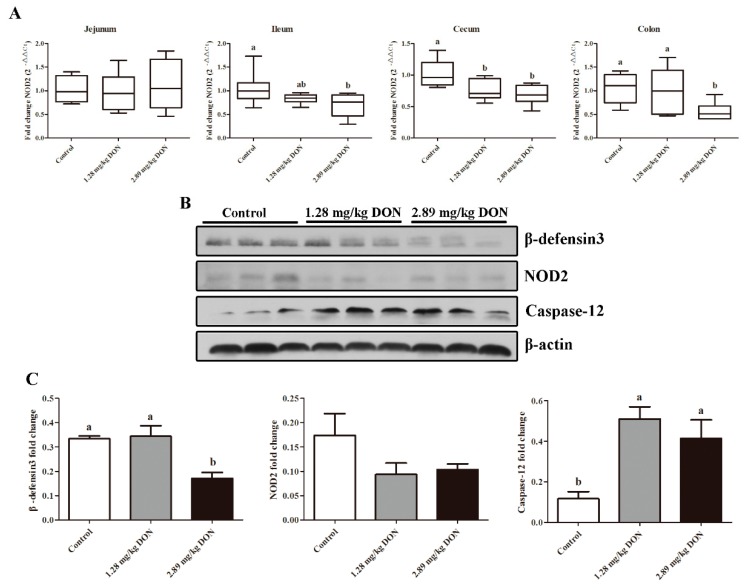
Modulating of caspase-12 was responsible for the depressed NOD2-mediated intestinal host defense peptides production induced by deoxynivalenol (DON). Effect of DON exposure on mRNA expression of NOD2 (**A**) in the jejunum, ileum, cecum, and colon (n = 8 animals). (**B**) Western blot analysis of β-defensin3, NOD2, and caspase-12 expression in jejunum. (**C**) Band intensity quantification of β-defensin3, NOD2, and caspase-12. Equal loading was assessed by β-actin immunoblotting. Values are means ± SEM, n = 3. ^a,b^ Mean values without a common letter were significantly different (*p* < 0.05).

NOD2 activation and resulting induction of HDPs constitute an early innate defense mechanism [10]. Firstly, we investigate the effect of DON on NOD2 mRNA expression in different intestinal segments. As shown in Figure 5A, the exposure to 2.89 mg/kg DON for 28 d decreased (*p* < 0.05) the NOD2 mRNA expression in ileum, cecum, and colon. As caspase-12 is an inhibitor of NOD2-mediated HDPs production, we next determined whether DON can enhance the expression of caspase-12 in jejunum. Western blot results showed that 2.89 mg/kg DON treatment led to a significant increase (*p* < 0.05) in caspase-12 protein level, with a consequent decrease (*p* < 0.05) in β-defensin3 protein abundance in jejunum. However, the abundance of NOD2 protein did not differ among the three groups (Figure 5B,C). 

### 2.6. Caspase-12 Was a Critical Regulator Modulating NOD2-Mediated HDPs Production in Response to DON Exposure

**Figure 6:**
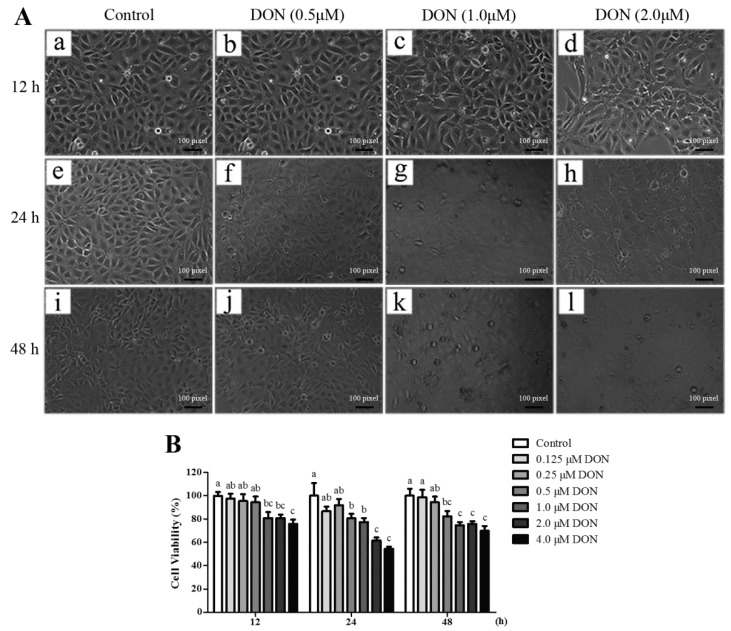
Effect of deoxynivalenol (DON) on the morphology and cell viability of IPEC-J2 cells. (**A**) Morphological observations of IPEC-J2 cells treated with various concentrations of DON for 12 h, 24 h, and 48 h. (**a**–**d**) IPEC-J2 cells were exposed to 0, 0.5, 1.0 or 2.0 μM DON for 12 h; (**e**–**h**) IPEC-J2 cells were exposed to 0, 0.5, 1.0 or 2.0 μM DON for 24 h; (**i**–**l**) IPEC-J2 cells were exposed to 0, 0.5, 1.0 or 2.0 μM DON for 48 h; (**B**) Cells were exposed to different concentrations of DON for 12 h, 24 h, and 48 h. At the indicated time, cell viability was determined using the cell counting kit-8 (CCK-8) reagent. Values are means ± SEM, n = 8. ^a,b,c^ Mean values without a common letter were significantly different (*p* < 0.05).

To determine the appropriated experimental concentrations of DON for subsequent stimulation experiments, the cytotoxic effects of DON on IPEC-J2 cells were assayed by measuring the cell viability (CCK-8 test) after exposure to various concentrations of DON for 12 h, 24 h, or 48 h. Morphological observation using phase contrast microscopy indicates that cells treated with DON show flat appearance, blurred boundary, and enhanced refraction (Figure 6A). As shown in Figure 6B, DON treatment led to decreased cell viability in a dose and time-dependent manner with statistically significant (*p* < 0.05) effects observed at 1.0 μM for 12 h, 0.5 μM for 24 h or 48 h, respectively. 

**Figure 7:**
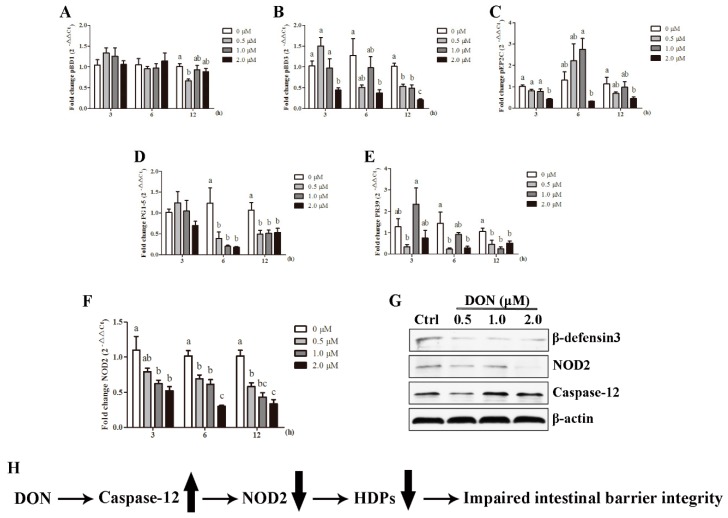
Caspase-12 plays critical role in modulating NOD2-mediated HDPs production in response to DON exposure. Expression of (**A**) pBD1, (**B**) pBD3, (**C**) pEP2C, (**D**) PG1-5, (**E**) PR39, as well as (**F**) NOD2 with exposure of DON in IPEC-J2 cells for 3 h, 6 h, and 12 h. Values are means ± SEM, n = 6. ^a,b,c^ Mean values without a common letter were significantly different (*p* < 0.05). (**G**) The immunoblot obtained with antibodies for β-defensin3, NOD2, caspase-12, and β-actin. (**H**) Proposed mechanism responsible for DON-induced HDPs dysregulation in the pathogenesis of impaired intestinal barrier integrity. Arrow pointing up indicates up-regulation, whereas arrow pointing down means down-regulation.

Levels of mRNA expression of HDPs in IPEC-J2 cells following exposure to DON are presented in Figure 7. The mRNA expression of pBD3, pEP2C, and PR39 were down-regulated (*p* < 0.05) in IPEC-J2 cells upon treatment with DON starting at 3 h and continuing to 12 h. The relative levels of pBD1 mRNA were significantly decreased (*p* < 0.05) after incubation with 0.5 μM DON for 12 h. In addition, cells exposed to 0.5, 1.0 and 2.0 μM DON for 6 and 12 h had lower (*p* < 0.05) PG1-5 mRNA levels compared with the control. 

To further explore the molecular mechanisms modulating the expression of HDPs in IPEC-J2 cells, we investigated the possible involvement of key regulators that are responsible for the HDPs production. Compared with the control group, the mRNA expression of NOD2 in DON-treated IPEC-J2 cells showed a significant decrease (*p* < 0.05) at 3 h, 6 h, and 12 h. Correspondingly, DON treatment led to decreased protein levels of β-defensin3 and NOD2 in IPEC-J2 cells. In contrast, the protein level of caspase-12 was elevated in response to DON stimulation (Figure 7G). 

## 3. Discussion

The gastrointestinal tract mucosa is continually exposed to a high load of antigens, ranging from dietary proteins and commensal microbiota to clinically important pathogens, viruses, and toxins. HDPs, produced by intestinal epithelial cells, constitute an effective anti-infection barrier in early response to microbial infections, inflammation, and tissue injury [22]. As the efficient defense components of the innate immunity, HDPs dysregulation would result in decreased disease resistance, posing a health hazard to pigs. Although the cytotoxic effects of DON on intestinal tissue of pigs have been well-studied, its role in disturbing the expression of intestinal HDPs remains largely unknown. Therefore, understanding the mechanism of DON-related intestinal HDPs dysregulation would provide important clues for future therapeutic strategies to prevent its adverse effects. 

The first zootechnical parameter affected by dietary exposure to DON (> 1 mg/kg in the feed of pigs) is depression in feed consumption, which consequently leads to reduced weight gain of piglets [23]. In the current study, declining ADFI and ADG reduction were observed in piglets that were fed a mono-contaminated diet with 1.28 mg/kg or 2.89 mg/kg of DON. Our results are consistent with previous studies, which showed that pigs that were fed diets contaminated with 1 to 2 mg/kg DON exhibited partial feed refusal and decreased weight gain [24,25]. 

In pigs, DON is rapidly and efficiently absorbed in the small intestine [26]. Intestine is considered to be the principal target for DON. The major histological changes in small intestine observed were villus fusion and atrophy, flattened enterocytes, and diffuse apical necrosis. Consistent with previous reports [2,27], we detected an obvious decline in villus height in duodenum and jejunum of pigs that ingested 1.28 mg/kg or 2.89 mg/kg DON-contaminated diet. Villus flattening in duodenum and jejunum is probably ascribed to the impairment of cell proliferation, as demonstrated by the *in vitro* cell viability assay. A shortening of the villi will result in poor nutrient absorption, diarrhea, and consequently lead to poor performance. To further parallel our findings, biomarkers associated with intestinal permeability were assayed. The content of endotoxin and concentrations of diamine oxidase in blood positively related to intestinal permeability [28]. Zonulin is a mediator known to modulate intestinal permeability via regulating intracellular tight junctions, which can be used as a potential marker to reflect intestinal permeability [21]. With impairment of intestinal barrier integrity, levels of serum endotoxin, diamine oxidase, and zonulin elevate [28]. In our study, significantly increased serum concentrations of endotoxin, diamine oxidase, and zonulin in pigs fed DON-contaminated diets further indicate that DON exposure impairs intestinal barrier function in weaned piglets. 

Host defense peptides are expressed at high concentrations at the intestinal mucosal surface, where they serve critical roles in maintenance of intestinal barrier integrity [4]. The intestinal epithelial cells secrete a rich arsenal of HDPs, such as defensins and cathelicidins. Thirteen β-defensins and eleven cathelicidins have been identified in pigs until now [29]. Porcine β-defensin 1, pBD2, pBD3, pBD129, and pEP2C are expressed in a broad range of tissues. Porcine cathelicidins including PR39, PG1–5, as well as PMAP-23, and PMAP-37, are mainly secreted by bone marrow myeloid cells [30]. Increasing evidence suggests that HDPs play crucial role in gut homeostasis by direct antimicrobial activity and stabilization of epithelial barrier integrity [31]. In the current study, it was found that oral DON exposure downregulates intestinal HDP expression in weaned piglets. In addition, these results were also observed in IPEC-J2 cells. Further regression analysis indicates that the jejunal pBD1, pBD3, and pEP2C gene expression appear to be more responsive to DON induced reduction in ADG and ADFI. Our work suggests that the toxicity of DON on piglets is related to the depressed intestinal HDPs expression levels. 

NOD2 has been shown to mediate the expression of gastrointestinal HDPs. It has been reported that the production of α-defensins is reduced in Crohn’s disease patients, especially those who have NOD2 gene mutations [13]. NOD2-deficient mice also had diminished α-defensins expression in the intestine [32]. Therefore, impairment of NOD2 function may lead to microbial homeostasis because of defective regulation of HDP expression, resulting in chronic enteric diseases. In our study, we found piglets fed 2.89 mg/kg DON-contaminated diet had lower NOD2 mRNA expression in ileum, cecum, and colon. The inhibitory effect of DON on NOD2 expression was also demonstrated by the present *in vitro* study. This result is consistent with data from human enterocytes [19]. 

NOD signaling activation and downstream HDPs expression form an initial innate defense mechanism, whereas caspase-12 negatively regulates this mucosal immune response [16]. Caspase-12 is highly expressed in intestinal epithelium. Under normal physiological conditions, caspase-12 is expressed in an inactive form. However, caspase-12 is specifically irritated by ER stress when the hosts encounter infection or toxins challenge. As we expected, oral DON exposure at 2.89 mg/kg DON markedly increased caspase-12 protein abundance, with a consequent reduction in β-defensin3 protein level in jejunum. Similar phenomenon was also demonstrated in the *in vitro* study. 

In summary, our study indicates that the activation of caspase-12, as a consequence of DON exposure, inhibits NOD-2 mediated HDPs production, which in turn impairs the intestinal barrier integrity of weaned piglets (Figure 7H). This study highlights the content of DON in animal feed should be strictly controlled under the maximum acceptable level (0.9 mg/kg DON in compound feed for pigs) recommended by European Commission Recommendation 2016/1319/EU. Moreover, our study provides a promising target for future therapeutic strategies to prevent the toxic effects of DON.

## 4. Methods and Methods

### 4.1. DON Production and Analysis

*Fusarium graminearum* strain W3008 was kindly provided by College of Plant Science and Technology of Huazhong Agricultural University, China. The strain was grown on potato dextrose agar at 28 °C for seven days to obtain mature spores. Three hundred grams of ground maize and fifty grams of rice, as well as 140 mL sterilized distilled water were added to a 1-litre conical flask, and then autoclaved at 121 °C for 20 min. Each flask was inoculated by *F. graminearum* containing 1 × 10^6^ spores/gram, and incubated at 28 °C and 85% relative humidity for 28 days. Finally, the mold contaminated sample in each flask was dried in an air oven at 65 °C overnight, mixed, and sampled for detection of DON content. The resulting moldy product was determined to contain approximate 300 mg/kg DON. DON content was determined using an AgraQuant^®^ DON ELISA Test Kit following the manufacture’s protocol (Romer Labs, Singapore). 

### 4.2. Animals and Experimental Design

All experimental procedures were approved by Scientific Ethics Committee of Huazhong Agricultural University on 7 August 2015. The project identification code is HZAURA-2015-006. A total of twenty-four 28-d-old barrows (Duroc × Landrace × Large White) were housed in individual metabolic cages, and randomly allocated to three dietary treatments (eight pigs per group): control diet (0.61 mg DON/kg feed); diet containing 1.28 mg DON/kg feed; and diet containing 2.89 mg DON/kg feed. The diets were artificially contaminated with this moldy product containing DON that were described above. The basal diet was formulated to satisfy nutritional requirements of starting pigs as proposed by National Research Council [33] and was supplied in mash form. The ingredients and chemical composition of the basal diet are presented in Table 2. All other mycotoxins, including fumonisin, aflatoxins, ochratoxin A, and T-2 toxin, in the final diets were below the limits of detection.

Piglets had *ad libitum* access to feed and water throughout the 28 day experiment period. Pigs were individually weighed at the beginning and on d 14 and 28 of the experiment. Feed consumption was measured for the periods of d 1 to 14 and d 15 to 28. These values were used to calculate ADG, ADFI, and G:F. 

The health status of pigs was recorded, and the occurrence of diarrhea for each piglet was visually evaluated two times a day (9 a.m. and 5 p. m.) using the method as previously described [34]. Fresh excreta were scored with the following scores: 0 = solid, normal; 1 = slight diarrhea, loose and soft feces; 2 = positively unformed, semi-liquid; and 3 = extremely watery and foamy diarrhea. The occurrence of diarrhea was defined as keeping fecal scores at 2 or 3 for two consecutive days. The incidence of diarrhea (%) was calculated as [(number of pigs with diarrhea × diarrhea days)/(total number of pigs × total experimental days)]× 100%.

### 4.3. Sample Collection

On d 14 and 28, blood samples were obtained from all 24 piglets via puncture of the jugular vein. Blood samples were collected in heparin-free vacutainer tubes. All samples were centrifuged at 5000× *g* for 15 min at room temperature. The serum was carefully separated and preserved at –80 °C until analysis.

After 28 days of dietary exposure to DON, all piglets were euthanized with sodium pentobarbital and samples were collected to measure intestinal morphology and expression of intestinal HDPs. Middle sections (3 cm) of duodenum, jejunum, and ileum were rinsed with 0.9% physiological saline and fixed in 4% paraformaldehyde for subsequent histological inspection. Three-cm mid-jejunum, mid-ileum, mid-cecum, and mid-colon were also immediately washed in 0.9% physiological saline and collected in Eppendorf tubes for quantitative real-time PCR. In addition, these mid-jejunum samples collected in the Eppendorf tubes were also used for Western blot analysis. Each tube was immersed in liquid nitrogen and preserved at –80 °C. 

### 4.4. Chemical Analyses

Crude protein, calcium, as well as phosphorus contents in the basal diet were analyzed in accordance with Association of Official Analytical Chemists [35] procedures. Amino acids except methionine, cystine and tryptophan were measured using ion-exchange chromatography with an automatic amino acid analyzer (Hitachi L-8900, Tokyo, Japan) after acid hydrolysis with 6 M HCl (reflux for 24 h at 110 °C). After oxidation with performic acid and subsequent hydrolysis with 6 M HCl, methionine and cystine were separated by Reverse-Phase HPLC (Agilent 1200, Santa Clare, CA, USA).

### 4.5. Determination of Endotoxin, Diamine Oxidase, and Zonulin in Serum

The serum endotoxin, diamine oxidase and zonulin concentrations were determined using commercially available porcine ELISA kits (Mlbio, Shanghai, China) according to the manufacture’s protocol. All procedures were carried out in duplicate. 

### 4.6. Small Intestine Morphology

Samples from each intestinal segment were dehydrated and embedded in paraffin. Transverse sections were cut at 5 μm and stained with hematoxylin–eosin for histomorphometry observation. Villus height and crypt depth were determined using an Olympus BX53 microscope (Olympus, Tokyo, Japan) with 40× magnification and analyzed with an Axiovision software. At least 8 intact, well-oriented villi and their related crypt were assessed per sample.

### 4.7. Cell Culture and Treatment

The IPEC-J2 cells were grown in DMEM/F12 medium supplemented with 10% FBS, 100 units/mL penicillin/streptomycin (Life Technologies, Grand Island, NY, USA), and 5 μg/L insulin-transferrin-sodium selenite (ITS) (Sciencell, San Diego, CA, USA) at 37^o^C in a humidified 5% CO_2_ atmosphere. Purified DON toxin (Sigma Aldrich, St. Louis, MO, USA) was dissolved in DMSO to prepare a 0.2 mg/mL stock solution, which was used for the subsequent cell culture experiments.

For cell viability assay, IPEC-J2 cells were seeded in a 96-well plate (2 × 10^4^ cells/well) and incubated for 24 h. Then, cells were treated with DON (0.125–4.0 μM) for 12 h, 24 h, and 48 h. At the indicated time, 10 μl CCK-8 solution (Dojindo, Kumamoto, Japan) was added into each well and preserved at 37^o^C for an additional 2 h. The absorbance was measured at 450 nm by a microplate reader (Multiskan MK3, Thermo Fisher Scientific, Waltham, MA, USA). Cell viability treated with DON was presented as a percentage compared to control (untreated). The morphology was also examined and images were captured under a light microscope connected with a digital camera (TSView 7, Tucsen, Fuzhou, China).

For stimulation experiments, IPEC-J2 cells (1 × 10^6^ cells/well) were pre-incubated on 6-well plates. After reaching 80% confluence, cells were treated with DON (0.5, 1.0, or 2.0 μM) for different times (3, 6, or 12 h). At the indicated time, IPEC-J2 cells were collected to examine the mRNA expression of intestinal HDPs.

To investigate the cell pathway, IPEC-J2 cells grown in 100-mm dishes were treated with DON (0.5, 1.0, or 2.0 μM) for 12 h. Then, the cell protein was extracted for immunoblotting.

### 4.8. Quantitative PCR

The intestinal tissues and cells were lysed in TRIzol (Takara, Dalian, China). Total RNA was extracted following the manufacture’s protocol. The quality and amount of total RNA were measured by gel electrophoresis and a Nano Drop 2000 spectrophotometer (Thermo Fisher Scientific), respectively. The first-strand cDNA was synthesized from the extracted RNA (1 μg) using a PrimeScript 1st strand cDNA Synthesis Kit (Takara, Dalian, China). Primers used in this study are listed in Table 3. Real-time PCR was conducted on a Bio-Rad CFX384 Real-Time PCR System with SYBR Green PCR Master Mix (Takara, Dalian, China). The relative amounts of mRNAs were normalized with the housekeeping gene β-actin and analyzed by the 2^–ΔΔCt^ method [36].

### 4.9. Western Blotting

The frozen jejunum tissues or IPEC-J2 cells were lysed in RIPA buffer supplemented with protease inhibitors (Applygen, Beijing, China). The lysed samples were centrifuged at 10,000× *g* for 10 min at 4 °C. Protein containing supernatant was collected and quantified with a BCA Protein Assay Kit (Pierce, Rockford, IL, USA). Fifty μg of proteins were electrophoresed on SDS polyacrylamine gels and electrotransferred to PVDF membranes (Millipore, Bedford, MA, USA). Membranes were blocked using 1 × TBST supplemented with 5% BSA (Sigma Aldrich, St Louis, MO, USA) for 2 h at room temperature. The membranes were incubated with corresponding primary antibodies (1:1000 dilution for overnight at 4 °C) against β-defensin 3 (Santa Cruz Biotechnology, CA, USA), NOD2, caspase-12, and β-actin (ABclonal, Wuhan, China). After washing of membranes with 1 × TBST, membranes were incubated with a secondary antibody (HRP-conjugated goat anti-rabbit IgG) (ABclonal, Wuhan, China) at a ratio of 1:10,000 dilution for one h at room temperature. The protein bands were detected via Western Blot Luminance Reagent (Applygene, Beijing, China) by an imaging system (Carestream, New York, NY, USA). 

### 4.10. Statistical Analysis

All data were analyzed by ANOVA using the general linear model (GLM) procedure of SAS (version 9.2, SAS Inst. Inc., Gary, NC, USA). Student-Newman-Keuls multiple range test was used to separate statistical differences among different treatments. Simple linear and quadratic relationships between piglet performance parameters and jejunal HDPs mRNA expressions were examined using regression procedures. A *p* value < 0.05 was considered significant. Data were presented as mean ± SEM. 

## Figures and Tables

**Table 1:** Effects of deoxynivalenol (DON) exposure on the growth performance and diarrhea incidence of weaned piglets (n = 8) ^1^.

Items	Control	1.28 mg/kg DON	2.89 mg/kg DON	SEM	*p*-Value
Intitial body weight (kg)	7.89	7.24	7.72	0.24	0.15
Body weight at day 14 (kg)	14.16 ^a^	12.11 ^b^	12.74 ^ab^	0.5	0.027
Body weight at day 28 (kg)	23.39 ^a^	20.43 ^b^	20.48 ^b^	0.67	0.007
Average daily gain (g)					
1–14 days	447	347	358	29.51	0.051
15–28 days	660 ^a^	594 ^ab^	553 ^b^	26.21	0.028
1–28 days	554 ^a^	471 ^b^	456 ^b^	22.67	0.013
Average daily feed intake (g/day)					
1–14 days	676 ^a^	552 ^b^	567 ^b^	34.95	0.041
15–28 days	1217 ^a^	1072 ^b^	1027 ^b^	44.68	0.018
1–28 days	946 ^a^	812 ^b^	798 ^b^	35.48	0.013
Gain: Feed					
1–14 days	0.66	0.62	0.63	0.03	0.527
15–28 days	0.55	0.55	0.54	0.02	0.8
1–28 days	0.59	0.58	0.57	0.02	0.774
Diarrhea score	0.22	0.61	0.63	-	-
Diarrhea incidence (%)	9.38	25.45	29.02	-	-

^1^ Each value is the mean ± standard error of eight replicates. ^a,b^ Means within a row without a common superscript are significantly different (*p* < 0.05).

**Table 2:** Formulation and nutrient levels of the basal diet (% as-fed basis).

Item	1–28 d
Ingredient (%)	
Maize	62.64
Soybean meal, dehulled (CP, 46%)	13.80
Concentrated soyabean protein	5.00
Fish meal (CP, 64%)	2.50
Whey powder	10.00
Soybean oil	2.00
Dicalcium phosphate	1.00
Limestone	0.80
Salt	0.80
_L_-Lys (78%)	0.33
_DL_-Met (98%)	0.20
Choline chloride	0.13
Zinc oxide	0.30
Mineral and vitamin premix ^1^	0.50
Nutrient composition (% analysed) ^2^	
Crude protein	20.71
Dry matter	89.02
Calcium	0.85
Phosphorus	0.65
Lys	1.48
Thr	0.89
Met + Cys	0.92
Calculated nutritional value	
Metabolic energy (kcal/kg)	3250

^1^ Provided per kg of complete diet: vitamin A, 12,000 IU; vitamin D_3_, 2,500 IU; vitamin E, 30 IU; vitamin B_12_, 12 μg; vitamin K_3_, 3 mg; choline chloride, 400 mg; nicotinic acid, 40 mg; pantothenic acid, 15 mg; Cu, 10 mg; Fe, 90 mg; Mn, 40 mg; Se, 0.3 mg; I, 0.35 mg. ^2^ Values are the means of a chemical analysis performed in duplicate.

**Table 3:** Sequence of the primers used for quantitative PCR.

Gene	Forward Primer (5’–3’)	Reverse Primer (5’–3’)
β-actin	TACACCGCTACCAGTTCGC	GCTCGATGGGGTACTTGAGG
pBD1	TGCCACAGGTGCCGATCT	TGCCACAGGTGCCGATCT
pBD2	GACTGTCTGCCTCCTCTC	GGTCCCTTCAATCTGTTG
pBD3	CCTTCTCTTTGCCTTGCTCTT	GCCACTCACAGAACAGCTACC
pEP2C	ACTGCTTGTTCTCCAGAGCC	TGGCACAGATGACAAAGCCT
PG1-5	GTAGGTTCTGCGTCTGTGTCG	CAAATCCTTCACCGTCTACCA
PR39	AGCAGTCCTCGGAAGCTAATC	GTCATTGGATGGGTTCAAGGT
PMAP23	GGATTATAGACCTGCTGTGGA	AGAACTCTTCCCTGTGTCTTG
PMAP37	GCTGTGTGACTTCAAGGAGAA	GAAATCTCCTGACACCCTCATT
NOD2	GAGCGCATCCTCTTAACTTTCG	ACGCTCGTGATCCGTGAAC
